# Rift Valley fever and *Brucella* spp. in ruminants, Somalia

**DOI:** 10.1186/s12917-021-02980-0

**Published:** 2021-08-21

**Authors:** Ahmed A. Hassan-Kadle, Aamir M. Osman, Mohamed A. Shair, Omar M. Abdi, Abdulkarim A. Yusuf, Abdalla M. Ibrahim, Rafael F. C. Vieira

**Affiliations:** 1 Abrar Research and Training Centre, Abrar University, Mogadishu, Somalia; 2 Vector-Borne Diseases Laboratory, Department of Veterinary Medicine, Universidade Federal do Paraná, Curitiba, Paraná Brazil; 3 Department of Slaughterhouses, Somali Meat Company, Mogadishu, Somalia; 4 Global One Health initiative (GOHi), The Ohio State University, Columbus, Ohio USA

**Keywords:** Neglected zoonotic diseases, RVF, *Brucella* spp., One Health

## Abstract

**Background:**

Fourteen-years after the last Rift Valley fever (RVF) virus (RVFV) outbreak, Somalia still suffers from preventable transboundary diseases. The tradition of unheated milk consumption and handling of aborted materials poses a public health risk for zoonotic diseases. Limited data are available on RVF and *Brucella* spp. in Somali people and their animals. Hence, this study has evaluated the occurrence of RVFV and *Brucella* spp. antibodies in cattle, goats and sheep sera from Afgoye and Jowhar districts of Somalia.

**Methods:**

Serum samples from 609 ruminants (201 cattle, 203 goats and 205 sheep), were serologically screened for RVF by a commercial cELISA, and *Brucella* species by modified Rose Bengal Plate Test (mRBPT) and a commercial iELISA.

**Results:**

Two out of 609 (0.3 %; 95 %CI: 0.04–1.2 %) ruminants were RVF seropositive, both were female cattle from both districts. Anti-*Brucella* spp. antibodies were detected in 64/609 (10.5 %; 95 %CI: 8.2–13.2 %) ruminants by mRBPT, which were 39/201 (19.4 %) cattle, 16/203 (7.9 %) goats and 9/205 (4.4 %) sheep. Cattle were 5.2 and 2.8 times more likely to be *Brucella*-seropositive than sheep (p = 0.000003) and goats (p = 0.001), respectively. When mRBPT-positive samples were tested by iELISA, 29/64 (45.3 %; 95 %CI: 32.8–58.3 %) ruminant sera were positive for *Brucella* spp. Only 23/39 (58.9 %) cattle sera and 6/16 (37.5 %) goat sera were positive to *Brucella* spp. by iELISA.

**Conclusions:**

The present study showed the serological evidence of RVF and brucellosis in ruminants from Afgoye and Jowhar districts of Somalia. Considering the negligence of the zoonotic diseases at the human-animal interface in Somali communities, a One Health approach is needed to protect public health.

## Background

Rift Valley fever (RVF) and brucellosis are important neglected zoonotic diseases with severe negative economic impact as they affect livestock productivity and survival, and threaten human health [1, 2]. By reducing the productivity of livestock, these diseases significantly lower the quantity and quality of animal products and consequently erode household nutrition, income and food security [3]. In Somalia, livestock represents 60 % of the gross domestic product (GDP) and plays an important role in poverty alleviation [4]. Despite the importance of livestock in the country and the health risk of infectious diseases on people and their animals, epidemiological data on zoonotic diseases are scarce in the country.

Rift Valley fever virus (RVFV) is a zoonotic transboundary vector-borne virus of the genus Phlebovirus in the family Phenuiviridae affecting primarily domestic ruminants and humans [5, 6]. It was first identified in 1931 in the Rift Valley of Kenya [6, 7], and has caused many documented outbreaks in humans and livestock in Africa including Somalia, and in Arabian Peninsula and some Indian Ocean Islands [5]. The RVF is a World Organization for Animal Health (OIE) listed disease due to its potential to cause human illness and deaths, high livestock abortions and deaths, and a setback to international livestock trade [8]. The disease is often linked to persistent heavy rainfall and flooding, which causes the emergence of infected mosquitoes, *Aedes* spp., which is already infected via transovarial transmission, and thus, lead to spread of the virus to animals and humans [6].

Previous studies have described outbreaks of RVF in Somalia and Kenya [9], and recent data on the seroprevalence of the disease has been described in Ethiopia [10]. However, routine surveillance for RVFV in Sub-Saharan African countries is limited and outbreaks are underreported [6]. A previous study on RVF in Saudi Arabia has found seroprevalence of 22.05 and 8.49 % in sheep and goats imported from Somalia, respectively [11]. However, data on RVF in Southern Somalia is lacking.

Brucellosis is a neglected bacterial zoonotic disease that severely hinders livestock productivity and human health [12, 13]. The disease is caused by *Brucella* spp., with *B. abortus* and *B. melitensis* of particular importance in ruminant and human cases. Transmission from animals to humans occurs mainly through the ingestion of infected dairy products and direct contact with an infected animal [12, 14].

Previous studies on serological evidence of brucellosis throughout sub-Saharan Africa have been reported [15, 16]. However, it remains a neglected disease in Somalia with few data available for ruminants, the majority dated before the Civil War in the country [17–20]. The reported *Brucella* spp. prevalence in the country is of 4 % in sheep, 4.9 % in goats [21] and 5.5 % in cattle [19].

After 20 years of crisis, Somalia is still politically unstable, famine and lacking state-of-the-art knowledge on zoonotic diseases. The tropical climate and prevailing tradition of unheated milk consumption, handling of aborted materials and reproductive excretions with bare hands favours disease spread. Herein, we aimed to evaluate the presence of antibodies specific to RVFV and *Brucella* spp. in cattle, goats and sheep from two important districts of Somalia.

## Materials and methods

**Fig. 1:**
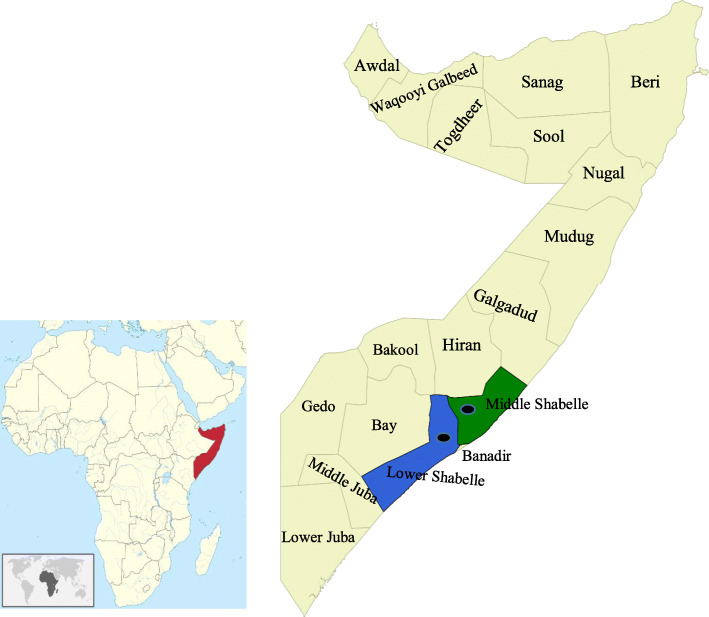
Map of Somalia showing the location of blood samples. Sampled regions are highlighted in green (Middle Shabelle) and blue (Lower Shabelle), and the dots indicate the approximate location of the sampled districts (Jowhar and Afgoye). The figure was generated and modified using QGIS software version 2.18.19

A total of 609 ruminant blood samples (201 cattle, 203 goats and 205 sheep) from Afgoye (2°08′47.67′′N 45°07′08.11′′E) and Jowhar (2°46′38.72′′N 45°30′05.85′′E) districts of Somalia (Fig. 1), collected from November 2017 to February 2018, previously surveyed for other pathogens [22] were evaluated. Blood and serum samples were kept at -20 °C for future studies. Serum samples were screened for RVFV by a commercial cELISA (ID Screen® Rift Valley fever Competition Multi-species, ID.vet, Grabels, France), which detects IgG antibodies specific to the RVFV nucleoprotein (NP) with 91–100 % sensitivity and 100 % specificity [23]. For *Brucella* spp., serum samples were initially tested by modified Rose Bengal Plate Test (mRBPT) with 89.6 % sensitivity and 84.5 % specificity [24]. The mRBPT positive samples were re-tested by a commercial indirect ELISA (iELISA) (ID Screen® Brucellosis Serum Indirect Multi-species, ID.vet, Grabels, France), which detects the IgG antibodies specific to *Brucella* Lipopolysaccharide (LPS) antigens with 96.8 % sensitivity and 96.3 % specificity [24]. The surveyed two districts are drained by Shabelle river and have suitable ecological conditions for vector breeding and disease occurrence. The cattle and small ruminants in these districts are kept under an extensive animal husbandry system and are often co-grazed.

Data were compiled and analyzed in Epi Info™ software, version 7.2.3.1 (Centers for Disease Control and Prevention, CDC, USA). Chi-square test was used to determine the difference between whether individual factors were associated with seropositivity to RVFV and *Brucella* spp. Odds ratio (OR), 95 % confidence interval and p-values were calculated separately for each variable. Results considered significantly different when p < 0.05.

## Results

Two out of 609 (0.3 %; 95 % CI: 0.04–1.2 %) ruminant sera showed positive reaction for RVF N protein, that were adult female cattle from Afgoye or Jowhar districts. Anti-*Brucella* spp. antibodies were detected in 64/609 (10.5 %; 95 % CI: 8.2–13.2 %) ruminants by mRBPT, which were 9/205 (4.4 %; 95 % CI: 2–8.2 %) sheep, 16/203 (7.9 %; 95 % CI: 4.6–12.5 %) goats and 39/201 (19.4 %; 95 % CI: 14.2–25.6 %) cattle. Cattle were more likely to be seropositive to *Brucella* spp. than sheep (OR: 5.2; χ2 = 21.9, p = 0.000003) and goats (OR: 2.8; χ2 = 11.4, p = 0.001). Association between districts (p = 0.453) or sex (p = 0.903) and seropositivity to *Brucella* spp. in ruminants was not found. When mRBPT-positive samples were tested by iELISA, 29/64 (45.3 %; 95 % CI: 32.8–58.3 %) ruminant sera also tested positive for *Brucella* spp. Only 23/39 (58.9 %) cattle and 6/16 (37.5 %) goat sera were reactive to *Brucella* spp. LPS antigens by iELISA. The seroprevalence of RVF and *Brucella* spp. for each variable evaluated is summarized in Table 1.

**Table 1:** Seroprevalence of RVFV and *Brucella* spp. in cattle, goats and sheep from Afgoye and Jowhar districts, Somalia 2017-2018

Variable	mRBPT – ***Brucella*** spp.				iELISA – ***Brucella*** spp.		cELISA – RVFV	
	+/***n***	Prevalence (%) (95% CI)	p value	OR (95% CI)	+/***mRBPT+***	Prevalence (%) (95% CI)	+/***n***	Prevalence (%) (95% CI)
**District**								
Afgoye	29/303	9.6 (6.5-13.5)	0.453 (*χ*^2^ = 0.6)	0.8 (0.5-1.4)	8/29	27.6 (12.7-47.2)	1/303	0.3 (0.01-1.8)
Jowhar	35/306	11.4 (8.1-15.6)			21/35	60 (42.1-76.1)	1/306	0.3 (0.01-1.8)
**Species**								
Cattle	39/201	19.4 (14.2-25.6)	0.000003 (*χ*^2^ = 21.9)	5.2 (2.5-11.1)	23/39	58.9 (42.1-74.4)	2/201	1 (0.1-3.5)
Goat	16/203	7.9 (4.6-12.5)	0.142 (*χ*^2^ = 2.2)	1.9 (0.8-4.3)	6/16	37.5 (15.2-64.6)	0/203	0.0
Sheep	9/205	4.4 (2.02-8.2)			0/9	0.0	0/205	0.0
**Sex**								
Female	59/559	10.6 (8.1-13.4)	0.903 (*χ*^2^= 0.02)	1.1 (0.4-2.8)	28/59	47.5 (34.3-60.9)	2/559	0.4 (0.04-1.3)
Male	5/50	10 (3.3-21.8)			1/5	20 (05-71.6)	0/50	0.0

+, number of positive animals; *n*, number of samples; 95% CI, 95% confidence interval; OR, odds ratio, mRBPT+, modified Rose Bengal Plate Test positive samples; iELISA, indirect ELISA; cELISA, competitive ELISA; RVF, Rift Valley fever

## Discussion

A limited number of studies on neglected zoonotic diseases have been reported in animals and humans in Somalia [22, 25]. Somalia is a livestock-dependent country in East Africa, which borders Kenya and Ethiopia where zoonotic transboundary diseases are often documented [6, 10].

To the best of the author’s knowledge, this is the first study on RVF in cattle, goats, and sheep in Afgoye and Jowhar districts of Somalia. Herein, overall, 1 % cattle were seropositive to RVFV by cELISA, and all goats and sheep tested negative. A previous study has reported high seroprevalence rates of RVFV in sheep (22.05 %, 30/136) and goats (8.49 %, 9/106) imported from Somalia to Saudi Arabia for pilgrimage season in 2011 [11]. It is important to state that neither the origin of the imported animals nor the quarantine status after arriving in Saudi Arabia was specified in that study. It was, therefore, impossible to affirm the origins of those animals. Moreover, a previous study in northern Somalia have found an overall RVF seroprevalence of 2 % in sheep and 5 % in goats sampled in Somaliland in 2001 and in Puntland in 2003 [26]. The last documented RVF outbreak occurred in 2007 in southern Somalia, specifically in the Middle and Lower Juba and Gedo regions both located at the border of Kenya, where the outbreak focus was reported [27].

In the present study, we evaluated ruminant’s serum samples from Afgoye and Jowhar districts, where RVF outbreaks have never been reported, collected between November 2017 to February 2018, which represents the dry season in Somalia [22] and may have influenced the low seropositivity found due to the low survival and proliferation of *Aedes* spp. Differing, a higher RVF prevalence has been found in sheep and goats from the Nugal Valley, Somaliland, in 2001 [26]. Our results are in line with the RVF findings obtained in Egypt (0 % in goats and 0.46 % in sheep) after 12 years from the last RVF outbreak in that country [28]. Thus, we hypothesize that animals evaluated by Mohamed et al. [11] may be the remnant livestock from the last documented RVFV outbreak in Somalia, which needs to be further investigated. Finally, a recent RVF outbreak has been confirmed in Isiolo, Mandera, Murang’a and Garissa counties of Kenya in February 2021 [29]. Thus, considering that free cattle movement between Kenya and Somalia occurs, RVF cases may also occur in Somalia, mainly during the rainy season.

Herein, the overall 10.5 % ruminants (19.4 % cattle, 7.9 % goats and 4.4 % cheep) were seropositive for *Brucella* spp. by mRBPT. Similar findings were found in Nigeria [30] and Sudan [31], in which cattle showed higher *Brucella* spp. seroprevalence rates than sheep and goats. It has been found that the prevalence of brucellosis is higher in pastoral grazing areas than in the urban and peri-urban areas [32]. In the present study, sheep and goats were originally from urban and peri-urban areas of Afgoye and Jowhar districts, which may explain the lower *Brucella* seroprevalence found. Moreover, previous studies on *Brucella* spp. in Somali cattle have found low seroprevalence rates of 0.7 % in Benadir region [20] and 5.5 % in Somaliland [19]. Differences in *Brucella* spp. prevalence may be due to variation in location, husbandry and management system, and serological test used (RBPT x mRBPT) [33]. Differences in the results of the two tests applied herein were due to the higher sensitivity and specificity of the commercial iELISA compared to the mRBPT.

In neighboring countries, such as Kenya, it has been pointed that there is a risk for re-emergence and transmission of brucellosis because of the co-existence of animal husbandry activities and social-cultural activities [34]. In fact, a strong association between human and animal *Brucella* seropositivity has been reported [35]. In Somalia, along with the same social-cultural activities there is a prevailing tradition of unheated milk consumption and handling of aborted materials and reproductive excretions with bare hands [36]. Thus, there is a risk of human cases of brucellosis particularly in groups occupationally or domestically exposed to those ruminants. Our data highlights the need for a One Health approach in the country aiming to reduce the odds of zoonotic transmission.

## Conclusions

The present study provides evidence for the serological prevalence of circulating antibodies against RVFV and *Brucella* spp. in ruminants in Somalia. Considering the negligence of the zoonotic diseases at the human-animal interface in Somali communities, there is a need to promote the One Health concept among multi-sectoral professionals and decision-makers for better and sustainable integrated health development and implementing effective control strategies against these zoonotic diseases.

## Data Availability

Not applicable.

## Abbreviations

ARTC: Abrar Research and Training Centre
AU: Abrar University
*Brucella* LPS: *Brucella* Lipopolysaccharide
CDC: Centers for Disease Control and Prevention
cELISA: Competitive Enzyme Linked ImmunoSorbent Assay
CI: confidence interval
CNPq: Conselho Nacional de Desenvolvimento Científico e Tecnológico
GDP: Gross Domestic Product
GOHi: Global One Health initiative
iELISA: indirect ELISA
IsDB: Islamic Development Bank
mRBPT: modified Rose Bengal Plate Test
OIE: World Organization for Animal Health
OR: Odds ratio
RVF: Rift Valley fever
RVFV: RVF virus
RVFV NP: RVFV nucleoprotein
TWAS: The World Academy of Sciences
UFPR: Universidade Federal do Paraná
UNESCO: United Nations Educational, Scientific and Cultural Organization
VBDL: Vector-Borne Diseases Laboratory
